# Parkinson’s Disease Motor Subtypes Show Different Responses to Long-Term Subthalamic Nucleus Stimulation

**DOI:** 10.3389/fnhum.2018.00365

**Published:** 2018-10-04

**Authors:** Cuiping Xu, Ping Zhuang, Mark Hallett, Yuqing Zhang, Jianyu Li, Yongjie Li

**Affiliations:** ^1^Beijing Institute of Functional Neurosurgery, Xuanwu Hospital, Capital Medical University, Beijing, China; ^2^Center of Parkinson’s Disease, Beijing Institute for Brain Disorders, Beijing, China; ^3^Key Laboratory of Neurodegenerative Diseases, Ministry of Education, Capital Medical University, Beijing, China; ^4^Human Motor Control Section, Medical Neurology Branch, National Institute of Neurological Disorders and Stroke, National Institutes of Health, Bethesda, MD, United States

**Keywords:** Parkinson’s disease, motor subtypes, deep brain stimulation, subthalamic nucleus, long-term effects

## Abstract

**Background and purpose:** Subthalamic nucleus deep brain stimulation (STN DBS) is well established for the treatment of advanced Parkinson’s disease (PD), substantially improving motor symptoms, quality of life, and reducing the long-term need for dopaminergic medication. However, whether chronic STN DBS produces different effects on PD motor subtypes is unknown. This retrospective study aimed to evaluate the long-term effects of STN DBS on the PD motor subtypes.

**Methods:** Eighty patients undergoing STN DBS were included. The Unified Parkinson’s Disease Rating Scale (UPDRS) analysis was performed in “On” and “Off” medication/“On” and “Off” stimulation conditions. The patients were classified as akinetic-rigid type (ART), tremor-dominant type (TDT), and mixed type (MT) based on the preoperative UPDRS III subscores in the “Off” medication state. Preoperative and postoperative comparisons were performed.

**Results:** After 4.9 years, STN DBS produced significant improvement in the UPDRS III total scores and subscores of tremor, rigidity, and bradykinesia in the “Off” medication state in the ART group, less improvement in the MT group, and the least improvement in the TDT group. The UPDRS II and III total scores and other subscores failed to improve during the “On” medication state. However, all groups improved substantially, and the improvement in tremor was sustained for both the “On” and “Off” medication states after years. Long-term STN DBS failed to improve swallowing and speech in all the subtypes.

**Conclusion:** The data confirms that PD is heterogeneous. Long-term STN DBS produced the best effects on bradykinesia/rigidity in the “Off” medication state and on tremor in the “On” and “Off” medication states. There were differences in the response by each group, but some of the differences could be explained by the fact that more severe symptoms at baseline tend to have greater improvement. The findings support the idea that ART mainly involves the basal ganglia-thalamo-cortical pathway, whereas TDT involves a different circuit, likely the cerebellar-thalamo-cortical pathway.

## Introduction

Subthalamic nucleus deep brain stimulation (STN DBS) is well established for the treatment of advanced Parkinson’s disease (PD), substantially improving motor symptoms, quality of life, and reducing the long-term requirement of dopaminergic medication (Krack et al., 2003; Deuschl et al., 2006; Benabid et al., 2009; Gervais-Bernard et al., 2009; Volkmann et al., 2009; Aviles-Olmos et al., 2014).

Parkinson’s disease is heterogeneous and can be classified into different subtypes (Hughes et al., 1992; Zaidel et al., 2009). Clinical and experimental data have indicated that patients with predominant akinesia/rigidity that is akinetic-rigid type (ART) versus those with predominant tremor that is tremor-dominant type (TDT) reflect different pathophysiologies (Hughes et al., 1992; Zaidel et al., 2009) and clinical courses (Paulus and Jellinger, 1991; Jankovic and Kapadia, 2001; Rajput et al., 2008). Patients with ART PD present more rapid progression and greater cognitive impairment than those with TDT PD (Jankovic et al., 1990). Long-term follow-up studies have demonstrated that parkinsonian symptoms respond variably to STN stimulation, showing improvement for some symptoms and deterioration for others (Rizzone et al., 2014). These studies mainly focused on the effects of STN DBS for PD motor symptoms, medication consumption, or other complications. Whether long-term STN DBS produces different clinical outcome on PD motor subtypes is unknown. In this study, we aimed to investigate the long-term effects of STN DBS on patients with TDT, ART, and mixed type (MT) PD subtypes. We hypothesized that patients with different subtypes would have different outcomes with long-term STN DBS.

## Materials and Methods

### Patients

Eighty-five consecutive patients (52 males, 33 females) with PD who were undergoing bilateral (*n* = 51) or unilateral (*n* = 34) implantation of STN DBS were studied. Of these, eighty were patients who had follow-up visits for more than 3 years. Five patients could not complete the 3-year follow-up visits owing to the fact that they were bed bound after stroke (*n* = 2) and had diabetes with severe peripheral neuropathy (*n* = 3) and were, therefore, not included in the present retrospective study.

All patients were diagnosed with PD according to the criteria of the UK Parkinson’s Disease Society Brain Bank (Hughes et al., 1992). Their mean age was 57.9 ± 9.6 years (range 31.0–75.6 years); mean disease duration was 8.3 ± 3.7 years (range 2–20 years); and mean L-dopa equivalent daily dose (LEDD) was 639.6 ± 314.0 mg/day. They were evaluated using the Unified Parkinson’s disease Rating Scale (UPDRS) (Fahn et al., 1987) and the Hoehn and Yahr scale (Hoehn and Yahr, 1967), while they were off their medications. Their mean UPDRS III score was 39.0 ± 17.2 and the Hoehn and Yahr scale score was 2.8 ± 0.7 at the time of surgery.

The subgroups of PD that include TDT, ART, and MT were classified by means of the UPDRS III using a method similar to that used by Lewis et al. (2005). First, a “tremor score” and a “non-tremor score” were calculated for each patient: the tremor score was derived from the sum of UPDRS item 20 (tremor at rest) and 21 (action and postural tremor of hands) divided by 7 (the number of single subitems included). The non-tremor score was derived from the sum of UPDRS item 18 (speech), 19 (facial expression), 22 (rigidity), 27 (arising from chair), 28 (posture), 29 (gait), 30 (postural stability), and 31 (body bradykinesia and hypokinesia) divided by 12 (the number of single subitems included). Patients were classified as TDT, if the tremor score was at least twice the non-tremor score. On the contrary, patients were classified as ART, if the non-tremor score was at least twice the tremor score. The remaining patients, in whom the tremor and non-tremor scores differed by less than a factor of 2, were classified as MT. In the three subtype groups, there were patients taking dopamine agonists such as Piribedil (50 mg/dosage) and Pramipexole (0.25 mg/dosage). However, there were no significant differences in the doses of dopamine agonist between the subtype groups either preoperatively or postoperatively during the “Off” and “On” states (ANOVA, *p* = 0.07–0.08).

The study was approved by the Ethics Committee of Xuanwu Hospital, Capital Medical University, China, according to the Declaration of Helsinki. Written informed consent was obtained from all patients.

### Surgical Procedure and Stimulation Programming

Details of surgical procedure have been previously described (Guo et al., 2013). Briefly, a standard stereotactic surgical procedure was performed using the CRW frame (Radionics, Burlington, MA, United States). The coordinates of anterior and posterior commissures were measured by using sagittal magnetic resonance imaging (MRI, Siemens 1.5 T, Sonata, Germany). Location of the STN was determined based on the stereotactic atlas of Schaltenbrand and Wahren (1977). The coordinates were as follows: 12 mm lateral, 1 mm posterior, and 4 mm inferior to the midcommissural point. Microelectrode recording was performed to determine the target of the STN. After the dorsal and ventral margins were determined, the longest and the most lateral segment of the STN was chosen to maximize the number of contacts within the STN as the ultimate DBS lead target. A quadripolar electrode (model 3389; Medtronic, Inc., Minneapolis, MN, United States) was implanted instead of a microelectrode. Efficacy and side effects were assessed using a test stimulator external control (mode 3625, Medtronic, Inc., Minneapolis, MN, United States). The internal pulse generators (Medtronic, Inc., Minneapolis, MN, United States) were implanted under general anesthesia on the day of surgery. The postoperative MRI was obtained 3–5 days after surgery to examine electrode position. Postoperatively, the contact with the best effect and the least adverse effect was chosen for stimulation (Guo et al., 2013).

All patients were off medications for at least 12 h (mean 13.3 ± 1.0 h, at range of 12–15 h) before surgery. They were requested to stay awake throughout the procedure to ensure their cooperation with neurosurgeons.

Stimulation was initiated within the first postoperative week, and optimal settings were selected. Later, stimulation and medication were further titrated based on clinical response over subsequent visits.

### Clinical Assessments

All patients were assessed preoperatively and postoperatively, which included assessments of motor function, speech, as well as quality of life that were carried out on the same day or within two consecutive days for each patient.

Motor function was evaluated using the UPDRS III. Patients were assessed in the “Off” state after overnight withdrawal of antiparkinsonian drugs and 2 h after the administration of levodopa in the “On” state.

After STN DBS, motor assessments were sequentially performed in the following conditions: “Off” medication/“On” stimulation (with stimulation switched on after 12 h medication withdrawal); and “On” medication/“On” stimulation (1 h after the administration of a suprathreshold dose of levodopa while stimulation was reintroduced). Subscores for individual cardinal features of PD were derived by the summation of the relevant items from the UPDRS III as follows: tremor (items 20–21), rigidity (items 22), bradykinesia (items 23–26, 31), and axial rigidity (18–19 and 27–30). The on state post-surgery assessment that was performed 1 h after the administration of medication was mainly according to the self-report of the patients. After 4–5 years, the patients were more advanced and the motor response was generally shorter.

Activities of daily living (ADL) was evaluated using the UPDRS II, and motor complications were evaluated using the UPDRS IV by including items 32–39 to assess dyskinesias and motor fluctuation. Functional performance was evaluated using the Schwab and England scale.

Since all patients kept their stimulation on continuously, postoperative data of the ADL and Schwab and England scales were only collected in the stimulation on condition, in the best on state and the practically defined “Off” medication state.

### Clinical Outcome Evaluation

The primary outcome measures were the scores of ADL scale and UPDRS III at baseline and at the clinical end points of 3–6 years. The secondary outcome measures were the subscores of UPDRS II (swallowing, writing, and freezing) and UPDRS III (tremor, rigidity, bradykinesia, speech, axial rigidity, postural stability, and gait), the scores on the Schwab and England scale of global ADL, and the dose of dopaminergic treatment at the last follow-up.

The motor improvement was calculated as follows: [(baseline “Off” or “On” medication score minus postoperative “Off” or “On” medication/“On” stimulation score)/baseline “Off” medication score] × 100 (positive scores denote improvement).

The change in score of motor response to DBS was calculated as follows: baseline “Off” or “On” medication score minus postoperative “Off” or “On” medication/“On” stimulation score (positive scores denote improvement).

### Medication

Each patient’s medication dose was recorded both preoperatively and postoperatively (at each time point), and LEDD was derived using a standard formula (Tomlinson et al., 2010). A stable level of medication was maintained for at least 2 months prior to surgery.

### Stimulation Parameters

Stimulation settings including the average voltage, pulse width, and frequency were calculated for all active contacts of each patient. The best contact was determined when it showed the best alleviation of the cardinal symptoms with the lowest voltage and without any side effects.

### Statistical Analysis

All data were expressed as mean ± standard deviation (SD). One-way ANOVA was used for clinical data of the three subtype groups. Furthermore, Bonferroni test was used for a two group comparison. For comparison between the baseline data and the data at the last follow-up after STN DBS, Student’s *t*-test was used. The X^2^ test was used for the comparison of non-parametric data in the three subgroups. All data were analyzed using SPSS version 19.0 (SPSS Inc., Chicago, IL, United States). Significance was set at *p* < 0.05.

## Results

The clinical characteristics and comparisons of three groups are demonstrated in **Table 1**. Patients with TDT, ART, and MT PD were not significantly different with respect to age, duration of disease, age at disease onset, dyskinesia, and LEDD (*p* > 0.05). Significant differences among the three groups were seen in scores of the Schwab and England scale and in the medication “On” and “Off” UPDRS II-III scores and subscores (all *p* < 0.01). The ART group presented the highest scores among the three groups.

**Table 1:** The demographic and baseline assessment of clinical characteristics of patients with three motor subtypes.

	TDT	ART	MT	*p*^a^	*p*^b^	*p*^b^	*p*^c^
Number	14	26	40				
Gender (M/F)	6/8	16/10	26/14				
Age at operation (y)	60.5 ± 6.4	55.5 ± 9.8	58.6 ± 10.3	0.246			
Age at disease onset (y)	52.2 ± 5.5	46.6 ± 10.2	50.7 ± 10.0	0.129			
Duration of disease (y)	8.3 ± 4.7	8.9 ± 4.6	8.0 ± 2.6	0.589			
Follow-up (y)	5.0 ± 1.2	4.9 ± 1.4	4.9 ± 1.3	0.994			
Hoehn and Yahr (Off)	2.2 ± 0.8	4.0 ± 1.1	3.3 ± 1.2	<0.001	<0.001**	0.004**	0.028*
Hoehn and Yahr (On)	1.4 ± 0.7	2.1 ± 0.9	1.8 ± 0.5	0.004	0.018*	0.053	0.097
Schwab and England (Off)	71.4 ± 15.6%	41.5 ± 26.0%	58.5 ± 22.1%	<0.001	<0.001**	0.04*	0.01*
Schwab and England (On)	85.0 ± 12.2%	76.5 ± 16.2%	84.8 ± 5.5%	0.011	0.036*	0.473	0.006**
UPDRS II (Off)	13.2 ± 7.9	21.8 ± 8.1	16.7 ± 8.5	0.006	0.001**	0.001**	0.016*
UPDRS II (On)	4.6 ± 4.8	8.4 ± 6.8	6.0 ± 2.6	0.035	0.018*	0.368	0.042*
UPDRS III (Off)	21.8 ± 9.8	48.3 ± 14.8	39.1 ± 16.2	<0.001	<0.001**	<0.001**	0.016*
UPDRS III (On)	8.5 ± 8.6	18.6 ± 10.9	13.9 ± 6.3	0.002	0.001**	0.042*	0.031*
Dyskinesia	0.9 ± 1.9	1.0 ± 1.7	0.5 ± 1.3	0.316			
L-Dopa response UPDRS III (%)	66.1 ± 26.2	62.3 ± 14.9	62.8 ± 13.1	0.764			
Rate of progression	6.1 ± 6.5	9.8 ± 5.4	7.9 ± 4.7	0.008	0.002**	0.024*	0.160
LEDD (mg)	646.4 ± 217.0	699.6 ± 429.4	598.3 ± 248.2	0.444			

*Off, Medication Off; On, Medication On; LEDD, L-dopa equivalent daily dose. ^a^indicates the interaction effect of the three groups (ANOVA); Bonferroni test indicates: ^b^all compared to TDT group, ^∗∗^p < 0.01; ^c^all compared to ART group, ^∗^p < 0.05. UPDRS, Unified Parkinson’s Disease Rating Scale; TDT, tremor-dominant type; ART, akinetic-rigid type; MT, mixed type.*

### Motor Outcome

#### “Off” Medication

Treatment with STN DBS significantly improved the UPDRS III scores for all patients with respect to baseline scores. Further analysis of the UPDRS scores and subscores showed that STN DBS produced significant and persistent effects on the ART and MT groups as compared with the TDT group. For the ART group, improvements were seen in the UPDRS III: total score = 46.6%; tremor = 80.6%; rigidity = 47.4%; bradykinesia = 45.3%; axial rigidity = 44.6%; gait = 44.8%; and postural stability = 56.0% (*p <* 0.001–0.007). For the MT group, improvements were seen in the UPDRS III: total score = 41.9%; tremor = 78.2%; rigidity = 46.8%; bradykinesia = 37.0%, axial rigidity = 10.3%; gait = 21.1%; and postural stability = 30.8% (*p* < 0.001–0.033, except for axial rigidity: *p* = 0.48; gait: *p* = 0.1). For the TDT group, a minor improvement was seen in the UPDRS III, total score = 5.0% (*p* = 0.45) and bradykinesia = 4.4% (*p* = 0.84), except for the significant improvement in tremor with 73.6% (*p* < 0.001). The other subscores of rigidity, speech, axial rigidity, gait, and postural stability deteriorated. Evidently, STN DBS produced a significantly positive effect on tremor but a negative effect on speech in all three subtypes of the PD patients.

**FIGURE 1:**
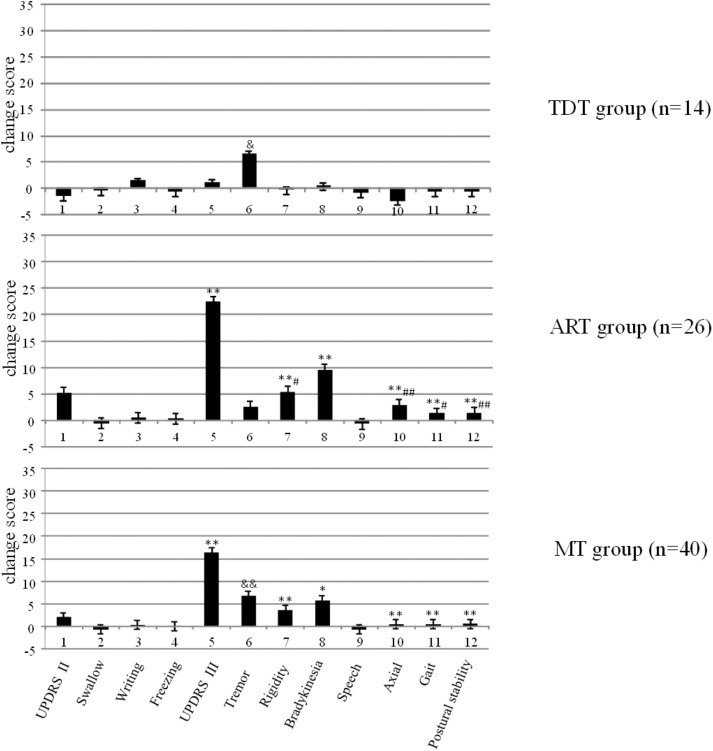
The changes in the UPDRS scores of TDT, ART, and MT patients during the “Off” medication state in response to long-term STN DBS. ^∗^compared with TDT, ^∗^*p* < 0.05; ^∗∗^*p* < 0.01; ^&^compared with ART, ^&^*p* < 0.05; ^&&^*p* < 0.01; ^#^compared with MT, ^#^*p* < 0.05; ^##^*p* < 0.01.

**Figure 1** demonstrates the changes in UPDRS II and III scores and subscores of the three groups and the significant differences that were observed within the groups (*p* < 0.003). Bonferroni test indicated that the ART group showed significantly higher improvement of UPDRS II and III scores and subscores than those of the MT and TDT groups.

**FIGURE 2:**
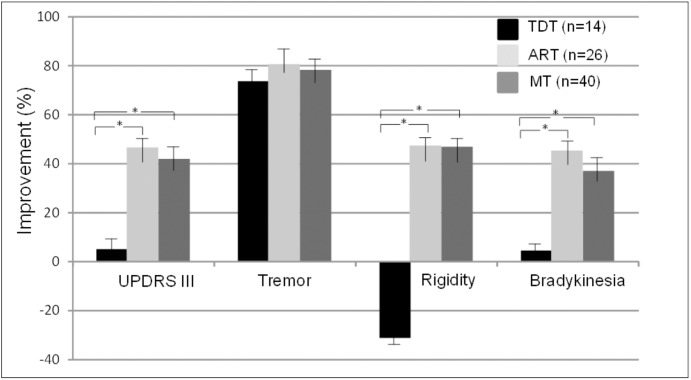
Comparisons of improvements in the UPDRS III scores and subscores across TDT, ART, and MT subtype groups during the “Off” medication state. ^∗^indicates TDT group compared with ART or MT groups (all *p* < 0.05, Bonferroni test).

**Figure 2** demonstrates the comparisons of the improvements in the UPDRS III subscores of tremor, rigidity, and bradykinesia across the three subtype groups. The ANOVA indicated that there were significant differences in the UPDRS III scores of rigidity and bradykinesia across the TDT, ART, and MT groups (*p* < 0.02–0.002). Bonferroni test showed that there were significant differences in the improvements in the UPDRS III scores of rigidity and bradykinesia in the TDT group when compared with those of the ART and MT groups. However, there were no significant differences in these score improvements between the ART and MT groups. In particular, there were no significant differences in tremor scores across the subtype groups (*p* = 0.79). The results suggested that the improvements of some subscores were likely independent of baseline assessment across the subtype groups.

**FIGURE 3:**
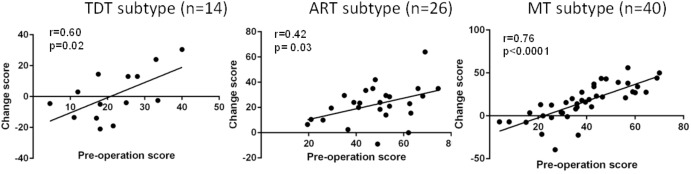
Correlation of the preoperative UPDRS III total score with the postoperative UPDRS III total score improvement (change score) across all groups during the “Off” medication state.

Overall, greater improvement was associated with more severe symptoms at baseline. **Figure 3** demonstrates the correlation data for the UPDRS III total scores across subtypes, suggesting that greater improvement is correlated with more severe symptoms at preoperative evaluation.

#### “On” Medication

Similar to recent findings of STN DBS studies, the UPDRS III total scores of the three subtype patient groups during stimulation on and medication on state did not change, but it deteriorated 4.9 years after surgery in comparison with the baseline clinical data. In addition to the significant improvement in tremor (80.8, 90.0, and 64.0%, *p* < 0.001–0.002) for the three groups and a minor improvement in rigidity for the ART and MT groups (8.7 and 11.1%, *p* = 0.48, 0.45), the subscores of bradykinesia, speech, axial rigidity, gait, and postural stability also deteriorated (**Table 2**).

**Table 2:** The UPDRS scores of three motor subtypes of patients evaluated pre- and post-surgery with a 4.9-year follow-up.

	Baseline			Mean 4.9 years ± 1.4 years(range of 3–6 years)			*p*-value		
	TDT (*n* = 14)	ART (*n* = 26)	MT (*n* = 40)	TDT (*n* = 14)	ART (*n* = 24)	MT (*n* = 40)	TDT	ART	MT
**“Off” medication**									
*UPDRS-II*	13.2 ± 7.9	21.8 ± 8.1	16.7 ± 8.5	14.9 ± 9.9	16.6 ± 8.2*	14.7 ± 8.0	0.656	0.015	0.337
Swallowing (item 7)	0.1 ± 0.3	0.5 ± 0.6	0.1 ± 0.3	0.7 ± 0.8*	1.1 ± 0.7**	0.8 ± 0.6**	0.038	0.004	<0.001
Writing (item 8)	2.1 ± 1.5	2.1 ± 0.9	1.7 ± 1.3	0.9 ± 0.7*	1.6 ± 1.0	1.5 ± 0.9	0.013	0.098	0.447
Freezing (item 14)	0.3 ± 0.6	2.0 ± 1.6	1.1 ± 1.3	1.2 ± 1.4	1.7 ± 1.2	1.2 ± 1.1	0.067	0.301	0.792
*UPDRS-III*	21.8 ± 9.8	48.3 ± 14.8	39.1 ± 16.2	20.7 ± 12.7	25.8 ± 15.9**	22.7 ± 13.5**	0.812	<0.001	<0.001
Tremor (items 20,21)	8.7 ± 4.5	3.1 ± 2.9	8.7 ± 5.2	2.3 ± 3.2**	0.6 ± 1.2**	1.9 ± 2.3**	<0.001	0.001	<0.001
Rigidity (item 22)	1.6 ± 2.0	11.4 ± 4.3	7.7 ± 4.1	2.1 ± 2.5	6.0 ± 3.9**	4.1 ± 3.2**	0.445	<0.001	<0.001
Bradykinesia (items 23–26,31)	9.0 ± 5.3	21.2 ± 8.0	15.4 ± 6.3	8.6 ± 6.4	11.6 ± 8.5**	9.7 ± 6.5**	0.844	<0.001	<0.001
Speech (item 18)	0.0 ± 0.0	0.6 ± 0.9	0.3 ± 0.6	1.1 ± 1.0**	1.2 ± 0.8**	1.1 ± 0.6**	0.004	0.007	<0.001
Other axial (items 19,27,28)	1.3 ± 1.2	6.5 ± 2.6	3.9 ± 2.3	3.9 ± 3.5*	3.6 ± 2.7**	3.5 ± 2.4	0.016	<0.001	0.483
Gait (item 29)	0.7 ± 1.0	2.9 ± 0.9	1.9 ± 1.0	1.5 ± 1.1	1.6 ± 1.1**	1.5 ± 1.0	0.106	<0.001	0.100
Postural stability (item 30)	0.5 ± 0.9	2.5 ± 1.3	1.3 ± 1.1	1.3 ± 1.1	1.1 ± 1.4**	0.9 ± 0.9*	0.067	<0.001	0.033
***“On” medication***									
*UPDRS-II*	4.6 ± 4.8	8.4 ± 6.8	6.0 ± 2.6	9.9 ± 9.1	11.8 ± 8.6*	10.2 ± 7.5**	0.084	0.020	0.001
Swallowing (item 7)	0.0 ± 0.0	0.4 ± 0.6	0.1 ± 0.2	0.5 ± 0.8*	0.9 ± 0.8*	0.5 ± 0.5**	0.038	0.010	<0.001
Writing (item 8)	0.9 ± 1.0	1.0 ± 0.6	0.6 ± 0.4	0.7 ± 0.6	1.2 ± 1.0	1.2 ± 0.8**	0.622	0.236	<0.001
Freezing (item 14)	0.0 ± 0.0	0.7 ± 0.7	0.5 ± 0.6	0.8 ± 1.4	1.1 ± 1.1*	0.7 ± 0.9	0.068	0.034	0.141
*UPDRS-III*	8.5 ± 8.6	18.6 ± 10.9	13.9 ± 6.3	14.4 ± 12.3	18.7 ± 14.7	15.1 ± 13.7	0.114	0.922	0.527
Tremor (items 20,21)	2.6 ± 2.4	1.0 ± 1.1	2.5 ± 1.8	0.5 ± 0.7**	0.1 ± 0.3**	0.9 ± 1.6**	0.002	0.001	<0.001
Rigidity (item 22)	0.7 ± 1.2	4.6 ± 2.4	2.7 ± 1.9	1.7 ± 2.3	4.2 ± 3.7	2.4 ± 2.6	0.104	0.484	0.457
Bradykinesia (items 23–26,31)	4.0 ± 5.1	8.1 ± 5.5	5.7 ± 3.0	6.8 ± 5.9	8.7 ± 7.7	6.6 ± 6.6	0.170	0.530	0.347
Speech (item 18)	0.0 ± 0.0	0.3 ± 0.5	0.2 ± 0.4	0.8 ± 0.9**	1.1 ± 0.9**	0.8 ± 0.6**	0.006	<0.001	<0.001
Other axial (items 19,27,28)	0.8 ± 0.9	2.7 ± 2.1	1.6 ± 1.0	2.8 ± 3.0*	2.7 ± 2.5	2.6 ± 2.3**	0.019	0.879	0.003
Gait (item 29)	0.3 ± 0.5	1.0 ± 0.6	0.7 ± 0.5	0.9 ± 1.1	1.2 ± 0.8	1.0 ± 0.9	0.064	0.221	0.080
Postural stability (item 30)	0.1 ± 0.4	1.0 ± 0.8	0.5 ± 0.5	0.9 ± 0.9*	0.8 ± 1.0	0.7 ± 0.9	0.026	0.143	0.361
*UPDRS-IV*									
Dyskinesias duration (items 32)	0.4 ± 0.9	0.5 ± 0.9	0.2 ± 0.5	0.6 ± 0.8	0.2 ± 0.5*	0.2 ± 0.4	0.317	0.020	0.739
Dyskinesias disability (items 33)	0.4 ± 0.9	0.5 ± 0.9	0.3 ± 0.9	0.7 ± 1.0	0.2 ± 0.6*	0.4 ± 0.8	0.317	0.020	0.765
Off duration (item 39)	1.9 ± 0.8	2.6 ± 0.9	2.2 ± 0.9	1.4 ± 0.5	1.7 ± 1.0**	1.7 ± 1.0*	0.089	<0.001	0.014

* and **indicate a significant difference (*p* < 0.05) and (p < 0.01), respectively, when compared with baseline. M-, without medication; M+, with medication; S-, without stimulation; S+, with stimulation; UPDRS, Unified Parkinson’s Disease Rating Scale. TDT, tremor-dominant type; ART, akinetic-rigid type; MT, mixed type.

**FIGURE 4:**
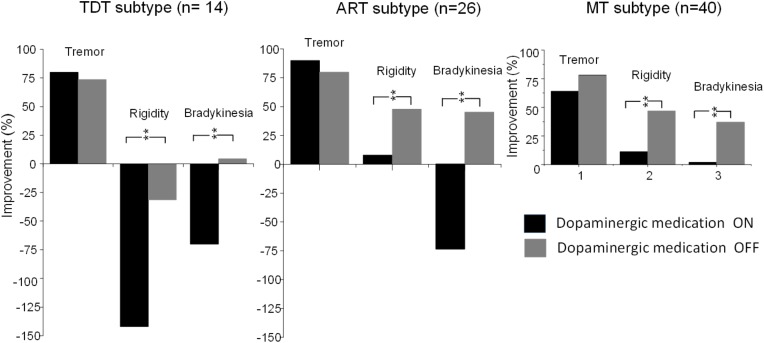
Comparisons of improvements in the UPDRS subscores of tremor, rigidity, and bradykinesia of TDT, ART, and MT groups during the “On” and “Off” medication states in response to long-term STN DBS. ^∗∗^indicates *p* < 0.01.

Compared with the UPDRS III subscores of rigidity and bradykinesia, long-term STN DBS produced sustained effects on tremor (all *p* < 0.001) in all subtype patients not only during the medication “Off” state but also during the “On” state (**Figure 4**).

### ADL

Compared with baseline, for ADL scores in the “Off” medication state, the ART group showed an improvement in the UPDRS II, total score = 23.9% (*p* < 0.02); writing = 23.8% (*p* = 0.098); and freezing = 15.0% (*p* = 0.30), but it deteriorated in the case of swallowing.

The TDT and MT groups showed improvements only in writing with 57.1% (*p* < 0.01) and 11.8% (*p* = 0.45), respectively.

The ADL score in the “On” medication state failed to improve except for a minor improvement in writing: 22.2% in the TDT group (**Table 2**).

### Drug-Related Motor Complications

For the three subgroups, significant improvements in L-dopa related motor complications were sustained over time with 4.9 years of stimulation as measured by the UPDRS IV subscores. Dyskinesia duration and dyskinesia disability was reduced by 60% (all *p* < 0.020) in the ART group. Off period duration was reduced by 34.6% (*p* < 0.001) in the ART group, by 22.7% (*p* < 0.014) in the MT group, and by 26.3% in the TDT group (**Table 2**).

### Medication Dosage

Postoperatively, the mean requirement for levodopa decreased. Percentages of LEDD decrease in the TDT, ART, and MT groups were 10.5, 6.0, and 15.9%, respectively.

At the last follow-up visit, 48 patients took only levodopa, 30 patients took a combined treatment, and 2 patients took benzhexol hydrochloride.

### Stimulation Parameters

Monopolar stimulation with the use of a single contact from the quadripolar electrode was applied in 68.8% of the patients. There were no significant differences between subgroups in voltage (TDT: 2.9 ± 0.5V; ART: 2.9 ± 0.4V; MT: 3.1 ± 0.4V; *p* = 0.092), frequency (TDT: 163.0 ± 20.9 Hz; ART: 174.2 ± 9.4 Hz; MT: 168.5 ± 13.4 Hz; *p* = 0.092); or pulse width (TDT: 86.0 ± 10.6 μs; ART: 87.9 ± 12.1 μs; MT: 87.2 ± 12.0 μs; *p* = 0.872).

### Active Contact Position

Of the 129 electrodes, 96 electrodes used monopolar stimulation and 33 electrodes used bipolar stimulation. In the 96 electrodes, the most active contacts were contacts 2/6.

### Adverse Effects

All patients received MRI after lead implantation. There were no hemorrhagic events, hardware infections in the primary procedure, or impulse generator replacements. There were no DBS malfunctions, lead fractures, or lead migrations.

Two patients had impulse control disorders in the first year post-surgery. One patient developed eyelid-opening apraxia 3 years post-surgery and was treated with botulinum toxin injections.

Five patients developed depression; three patients suffered moderate depression; one patient presented suicidal tendency; and one patient attempted self-mutilation. All patients with depression required antidepressant medication.

## Discussion

This study reported the long-term outcome of STN DBS for PD motor subtypes. The first important finding was that long-term STN DBS in the “Off” medication condition produced the best effects on bradykinesia/rigidity. Another important finding is that long-term STN DBS produces sustained effects on tremor score in all subtypes not only during the “Off” medication state but also during the “On” medication state. These findings strongly support the view that PD is a heterogeneous disease. ART likely involves the basal ganglia-thalamo-cortical pathway, whereas TDT likely involves other circuits, possibly the cerebello-thalamo-cortical pathway (Lewis et al., 2011).

Consistent with previous findings, our findings confirmed the beneficial anti-parkinsonian effects of long-term STN DBS on the UPDRS total scores and subscores of rigidity and tremor during the “Off” medication state. Improvement in dyskinesia was also maintained with long-term stimulation.

The UPDRS II and III total scores and subscores of rigidity, bradykinesia, axial rigidity, and gait motor symptoms during the medication “On” state remained unchanged initially but deteriorated after 4.9 years. Improvement in ADL was seen in ART patients during the “Off” medication state, but it worsened during the medication “On” state in all patients (Lang and Obeso, 2004). These findings suggest that STN DBS failed to improve swallowing and speech but led to their deterioration in all patients during the “On” and “Off” medication states. The worsening might be due to the natural progression of disease, most likely non-dopaminergic deficits.

In the current study, age, disease duration, age at disease onset, dyskinesia, and LEDD matched groups of ART, TDT, and MT PD patients did not differ significantly at baseline. However, significant differences were seen between subgroups in scores on the Hoehn and Yahr scale, Schwab and England scale, and UPDRS II and III subscores during the medication “On” and “Off” states. The ART group presented the highest scores among the three groups. After chronic STN stimulation, however, the greatest improvement in UPDRS II and III total scores and subscores of rigidity and bradykinesia were maintained in the ART group among the three groups during the “Off” medication state besides tremor. In contrast, STN DBS produced a sustained effect on tremor subscore during the “On” medication state. These findings strongly suggested that ART patients differed from TDT patients.

In this study, we also examined the possible impaction of the significant differences at baseline for certain measures between groups for the results. The analysis does show some results in accordance with the assumption that more severe symptoms improve in a clinical outcome. This is true for all measures in the MT group. However, for the TDT and ART groups, this factor influences only the tremor score and not the important manifestations of rigidity and bradykinesia. We believe that the subtype has influence on outcome.

In addition to the TDT and ART subtype groups, the present study had a large number of patients characterized into a mixed subgroup with equally shared features of the two symptom complexes. The presence of a mixed subgroup is not surprising in view of the progressive and degenerative nature of the disease and the selection bias in the current sample. These PD patients were surgical candidates, individuals who were by definition medically refractory and well into the course of their disease. Although there is no consensus, the recent reports suggest that approximately one-third of the total population of PD patients show predominant features of akinetic-rigid syndrome. Early in the disease, the breakdown process favors a tremor dominant majority over the akinetic-rigid or non-tremor dominant types. However, with the progression of the disease, many tremor-dominant individuals begin to emerge with more features of rigidity and bradykinesia, constituting a “mixed group.” The long-term STN DBS also showed a significant effect based on the subtype.

There is evidence that tremor is pathophysiologically disconnected from bradykinesia and rigidity (Hallett, 2012). Clinically, the manifestations are separate, and response of tremor to dopaminergic agents is less certain than bradykinesia (Hallett, 2014). Hoehn and Yahr (1967) first demonstrated that marked clinical diversity exists in PD, and later studies (Paulus and Jellinger, 1991; Mariama-Lyons and Koller, 2000; Spiegel et al., 2007; Rajput et al., 2008) provided experimental and clinical support for the PD subgroup differences. Further factor analysis of PD signs showed that rest tremor was relatively independent of other cardinal signs of PD, was less reliably responsive to dopaminergic modulation (Mariama-Lyons and Koller, 2000), and did not worsen at the same rate as bradykinesia and rigidity (Jankovic and Kapadia, 2001; Zaidel et al., 2009). ART patients present a faster clinical progression with more severe cognitive decline and worse prognosis, whereas TDT patients show a slower disease progression with less cognitive decline and better prognosis (Jankovic and Kapadia, 2001; Zaidel et al., 2009). Imaging studies demonstrate that the outcome is more favorable in TDT patients than in ART patients (Rajput et al., 2008). The ART PD patients have a distinct reduction in dopaminergic uptake associated with symptom progression (Vingerhoets et al., 1997; Eggers et al., 2012). Altogether, these findings support the view that dopamine depletion in the basal ganglia is more important in akinesia/rigidity (Zaidel et al., 2009) than in tremor (Spiegel et al., 2007; Hallett, 2014).

Currently, there is a strong hypothesis that parkinsonian tremor is mediated by linking two distinct circuits: the basal ganglia, which are primarily affected by dopamine depletion in PD, and the cerebello-thalamo-cortical circuit, which is also involved in many other tremors (Hutchison et al., 1997; Helmich et al., 2011; Milosevic et al., 2018). Specifically, the “dimmer-switch model” explains tremor as resulting from the combined action of the basal ganglia to trigger tremor episodes and the cerebello-thalamo-cortical circuit to produce tremor (Helmich et al., 2012). Contrastingly, it appears that bradykinesia and rigidity are related only to the basal ganglia pathways (without the cerebellar circuits).

There are several limitations to this study. Major limitations include the small sample size and the retrospective nature of the study. Another limitation is that we only focused on motor functions and failed to evaluate the cognitive functions. Common long-term adverse events after STN DBS comprise depression and progressive cognitive decline, which may lead to dementia. No doubt, further investigation will be carried out.

## Author Contributions

PZ and CX contributed to collect data, analysis and interpretation of the data, and drafting and revising the manuscript. MH contributed to the design, conceptualization of the study, and revising the manuscript. YZ, JL, and YL contributed to data collection and conceptualization of the study.

## Conflict of Interest Statement

The authors declare that the research was conducted in the absence of any commercial or financial relationships that could be construed as a potential conflict of interest.
